# Locoregional recurrence and survival of breast−conserving surgery compared to mastectomy following neoadjuvant chemotherapy in operable breast cancer

**DOI:** 10.3389/fonc.2024.1308343

**Published:** 2024-03-28

**Authors:** Fa-you Lv, Zongming Mo, Binjie Chen, Zhen Huang, Qinguo Mo, Qixing Tan

**Affiliations:** ^1^ Department of Breast Surgery, Guangxi Medical University Cancer Hospital, Nanning, Guangxi, China; ^2^ Department of Breast Surgery, Guangxi Zhuang Autonomous Region People’s Hospital, Nanning, Guangxi, China

**Keywords:** breast cancer, neoadjuvant chemotherapy, breast-conserving surgery, mastectomy, prognosis

## Abstract

**Background:**

The risk of locoregional recurrence (LRR) and the long-term prognosis of breast-conserving surgery (BCS) after neoadjuvant chemotherapy (NAC) are still controversial. This study aimed to evaluate oncological outcomes for patients undergoing BCS after NAC and determine LRR and survival predictors.

**Methods:**

This study was a retrospective cohort study of patients with locally advanced breast cancer (LABC) who received NAC and underwent BCS or mastectomy from June 2011 to November 2020. LRR, disease-free survival (DFS), and overall survival (OS) were compared in patients undergoing BCS or mastectomy. Univariate and multivariate analyses were performed to determine LRR, DFS, and OS predictors.

**Results:**

A total of 585 patients were included, of whom 106 (18.1%) underwent BCS and 479 (81.9%) underwent a mastectomy. The LRR rate was 11.3% in the BCS group and 16.3% in the mastectomy group, revealing no significant difference(*p* = 0.200). In patients who underwent BCS, clinical lymph node status, histological grade and pathological complete response (pCR) were independent factors to predict LRR. There was no significant difference in DFS and OS between the BCS and the mastectomy groups. Multivariable analysis showed that lymph node status, histological grade, molecular subtypes, pCR and Miller&Payne (M&P) classification were independent predictors of DFS. Lymph node status, molecular subtypes and pCR were independent predictors of OS. BCS or mastectomy was not an independent predictor of DFS or OS.

**Conclusion:**

Compared with mastectomy, BCS after NAC may not increase the risk of local recurrence or mortality, BCS can be performed in selected patients with small tumor size and good response to NAC.

## Introduction

Breast cancer is the most common malignancy in women worldwide (1). Survey results show that in developed countries, patients diagnosed with locally advanced breast cancer (LABC) at initial diagnosis account for 7% of the total number of breast cancer patients (2), while in developing countries, the proportion of LABC is as high as 30%-60% (3). Patients with LABC are likely to receive neoadjuvant chemotherapy (NAC) in current practice (4). NAC could reduce the primary lesions and metastatic lymph nodes, thus improve the resection rate of radical surgery (5, 6). NAC could also increase breast-conservation in woman initially scored as being appropriate for mastectomy. In addition, disease-free and overall survival rates were further improved in patients who achieved complete pathological response (pCR) after NAC (7). However, a multidisciplinary approach in breast cancer patients undertaking NAC may necessary to ensure the optimal outcome (8).

For surgical treatment after NAC, mastectomy and breast-conserving surgery (BCS) are commonly used in clinical practice. BCS could reduce the scope of surgical resection as far as possible, based on the complete resection of the lesion while retaining the cosmetic aspect of the breast, which is favored by patients. Some studies (9, 10) have shown that the long-term survival of patients undergoing BCS and postoperative radiotherapy is roughly the same as that of patients undergoing mastectomy, but the cosmetic effect of BCS is greatly improved. Traditionally, breast cancers with large tumors or special locations were not suitable for BCS. However, this view has changed with the application of NAC and oncoplastic surgery (OPS) techniques (11). OPS is a form of breast conservation that combines definitive oncologic resection with optimal aesthetic outcomes. In contrast to simple conservative surgery, OPS uses volume displacement techniques to close the lumpectomy defect and redistribute the remaining breast volume over the preserved breast. Thus, one of the benefits of OPS is the ability to remove larger specimens with less aesthetic impact. By downstaging the tumor, NAC can convert patients who are candidates for mastectomy to BCS candidates, especially for patients with centripetal tumor retraction and non-multicentric lesions. Furthermore, it can reduce excision volumes in patients with large cancer who are already candidates for BCS, improving cosmetic outcomes.

NAC has improved the rate of BCS for breast cancer patients and their postoperative quality of life has also been improved. However, the safety of BCS after NAC has not been determined. In the NSABP B-18 trial, the ipsilateral breast tumor recurrence (IBTR) rate of breast-conserving patients after NAC showed an increasing trend, but the difference was not statistically significant. A meta-analysis of outcomes after NAC suggested that BCS after NAC may lead to an increased LRR rate. The 15-year LRR rate of breast-conserving patients after NAC was 5.5% higher than that of patients receiving adjuvant chemotherapy (21.4% vs. 15.9%, respectively) (5). It is believed that the influencing factors are mainly related to the location of the tumor, the evaluation of regression and the condition of the surgical margin, etc. (5). By contrast, two recent retrospective studies have shown that BCS does not impair LRR and the long-term prognosis in patients treated with NAC (12, 13). It is believed that the influencing factors are mainly related to the location of the tumor, the evaluation of regression and the condition of the surgical margin, etc. (5). There are also studies (14, 15) showing that the recurrence rate of BCS after NAC is closely related to the size of the primary tumor. Therefore, the available data is controversial about whether BCS after NAC increases the risk of recurrence, and further studies are needed.

This study aimed to determine whether patients who received NAC have equal surgical outcomes after BCS therapy compared to mastectomy, and identify the predictors of local recurrence and survival to provide further insight into the feasibility and safety of BCS after NAC.

## Materials and methods

### Patient population

This study was a retrospective cohort study of patients with breast cancer who received BCS or mastectomy following NAC from June 2011 to November 2020, at Guangxi Medical University Cancer Hospital. Patients who met the following inclusion criteria were selected consecutively: (1) Female, over 18 years old; (2) Patients with clinical stage T1-4N1-2M0; (3) At least 4 cycles of NAC were completed; (4) No other anti-cancer therapy was performed prior to NAC; (5) Patients underwent breast surgery after NAC. Patients were excluded if they met the following exclusion criteria:(1) Patients who had other malignant tumors; (2) Bilateral breast cancer; (3) Distant metastasis occurred on admission;(4) Incomplete clinicopathological data or follow-up information;(5) Tumor resection was performed prior to NAC. The clinical characteristics, chemotherapy regimen, type of surgery, pathological outcomes, and follow-up information were collected. The study was conducted in accordance with the 1964 Helsinki declaration and its later amendments or comparable ethical standards, we have deidentified all patient details. The reporting of this study conformed to STROBE guidelines (16).

### Treatment

The patients in our cohort who received NAC regimens were determined based on the NCCN breast cancer guideline (17) and based on recommendations from the Chinese Society of Clinical Oncology (CSCO) (18). Core needle biopsy was performed on breast tumors and ipsilateral axillary lymph nodes before NAC, breast tattooing techniques are used to delimit the initial tumor size and its margins, and a clip is placed to mark the primary tumor site, if the surgical protocol is planned to be BCS. For patients with negative axillary lymph node biopsies, a clip is placed to mark the sentinel lymph nodes under ultrasound guidance. chemotherapy regimens included anthracycline-based and taxane-based regimens, and neoadjuvant trastuzumab treatment was administered to HER-2 patients. Breast magnetic resonance imaging (MRI) were performed as standards for response evaluation every two cycles of chemotherapy to evaluate the curative effect as per Recist 1.1. After the completion of NAC, all patients received surgical treatment. The surgical method was determined according to the clinician’s evaluation and the patient’s wishes, including mastectomy ( ± breast reconstruction) and breast-conserving. Mastectomy patients had subcutaneous mastectomy, total mastectomy or radical mastectomy. Axillary surgery consisted of sentinel lymph node biopsy (SLNB) for node-negative patients pre-NAC or axillary lymph node dissection for node-positive patients pre-NAC or patients with a positive SLNB. BCS was recommended for patients who meet the following criteria: 1) The tumor size after NAC was T1 or T2; 2) The tumor size>5cm, but the breast has an appropriate volume, and good breast shape can be maintained after BCS. Breast-conserving was performed by conventional BCS techniques or OPS techniques, techniques were chosen according to tumor localization and tumor-to-breast volume, thus ensuring tumor-free margins and achieve a good cosmetic outcome. According to the current expert consensus, level I oncoplastic surgery is defined as a resection of <20% of the breast volume, whereas the lumpectomy is closed by redistribution of ipsilateral breast tissue. Level II oncoplastic surgery is defined as a resection of 20–50% of the breast tissue (including circumvertical mastopexy design and reduction mammoplasty). The procedures of oncoplastic surgery for breast cancer is described in the references (19, 20). Frozen biopsies were analyzed to confirm clear tumor margins, and postoperative immunohistochemical pathology was examined again. Breast-conserving patients all received postoperative radiation therapy, while radiation therapy was given to patients with mastectomy for the following conditions, including tumor T3-4, axillary lymph node metastasis, tumor ≤5 cm and negative margins but <1 mm.

### Pathology and efficacy evaluation

Estrogen receptor (ER), progestogen receptor (PR) status, and human epidermal growth factor receptor 2 (HER-2) status were determined by immunohistochemical analysis, which was performed with formalin-fixed, paraffin-embedded tissue sections using standard protocols for core needle biopsy specimens by the pathology department of Guangxi Medical University Cancer Hospital. Pathological diagnosis of positive cells ≥1% is defined as positive for ER and PR; immunohistochemical HER-2 strong positive (+++) or Fish test prompts gene amplification is defined as HER-2 positive (pathological diagnosis is completed by two independent pathologists). Surgical specimens after NAC were pathologically evaluated according to the Miller-Payne grading system as described previously (21), and were divided into five grades through paired specimen examination before and after chemotherapy: Grade 1(G1), the number of tumor cells did not decrease in general; Grade 2(G2), no more than 30% reduction of tumor cells; Grade 3(G3), 30%~90% reduction of tumor cells; Grade 4(G4), significantly more than 90% reduction of tumor cells; Grade 5(G5), no invasive cancer cells were found in the tumor bed section, but ductal carcinoma *in situ* could exist. Pathologic complete response (pCR) is defined as no residual invasive cancer or only residual carcinoma *in situ* in the primary area and regional lymph nodes after chemotherapy.

### Follow-up

Follow-up information was collected through the outpatient service, telephone, and review, until death or the date of the last follow-up (on October 30, 2021). The whole group of patients’ median follow-up time was 43 months (8~135months). Locoregional recurrence was defined as the recurrence of tumors in the ipsilateral breast after breast-conserving surgery, in the ipsilateral chest wall after mastectomy, or in the patient’s lymph drainage area, including the axilla, internal mammary, and supraclavicular region. Disease-free survival was defined as the time interval from the date of breast cancer surgery to the date of evidence of local or distant recurrence, and overall survival was defined as the time interval from the date of breast cancer diagnosis to the date of death from any cause.

### Statistical analysis

SPSS 26.0 software (SPSS Inc., Chicago, IL, USA) was used for statistical analysis. Continuous and categorical variables were compared using the Mann–Whitney U-test and χ^2^ test, respectively. Survival curves between the two groups were compared by the Kaplan–Meier method and analyzed using the log-rank test. Univariable and multivariable Cox proportional hazards methods were used to evaluate factors predictive of LRR, DFS and OS. Variables with *p* ≤ 0.1 in the univariate analysis were candidates for multivariable analysis. Two-tailed *p* value < 0.05 was deemed statistically significant.

## Results

### Patient characteristics

**Figure 1:**
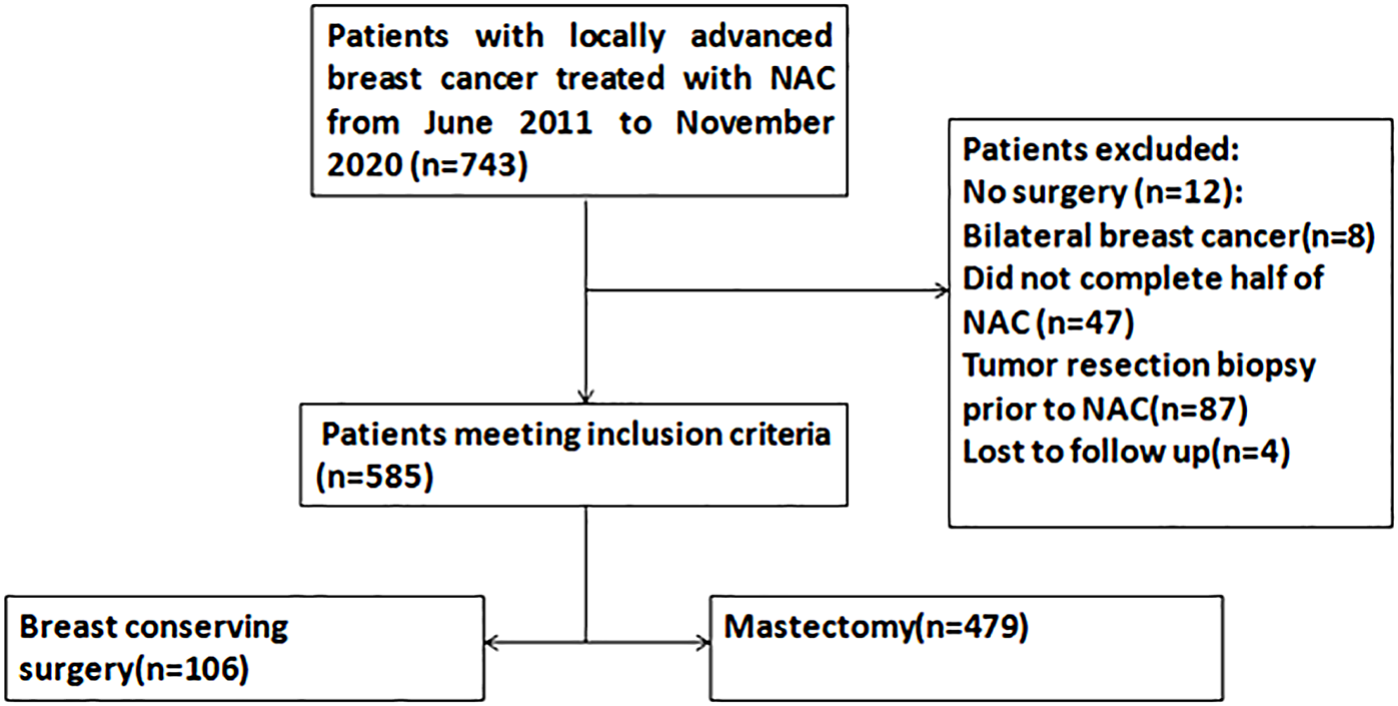
Flowchart of study selection process. T, tumor stage; N, nodal stage; M, metastasis; NAC, Neoadjuvant chemotherapy.

In total, 743 patients with stage II-III breast cancer who received NAC and underwent surgery were identified. 158 patients were excluded because of not undergoing surgery, bilateral breast cancer, not completing scheduled NAC, tumor resection before NAC, or loss to follow-up. Eventually, a total of 585 patients were eligible for analysis. The flowchart of the patient selection process is demonstrated in **Figure 1**. Among the 585 patients, 532 patients (90.9%) have received all chemotherapy before surgery, 106 patients (18.1%) received BCS and 479 patients (81.9%) received mastectomy. For axillary surgery, 118 patients (20.2%) received sentinel lymph node biopsy, of which 32 patients with positive sentinel lymph nodes underwent axillary lymph node dissection, and 467 patients (79.8%) received the axillary nodal dissection directly. After NAC, imaging assessment showed that 110 cases had complete response (CR), 367 cases had partial response (PR), 90 cases had stable response (SD), and 18 cases had progressive response (PD), the objective response rate (ORR) was 81.5%. 114 patients (19.5%) achieved pCR, patients who underwent BCS had higher pCR rates than those who underwent mastectomy (38.7% versus 15.2%, *p* < 0.001). The proportion of patients with multifocal or multicentric lesions in the mastectomy group was significantly higher than that in the BCS group (34.5% versus 15.1%, *p* < 0.001). The T stage pre-NAC in the BCS group were lower than those in the mastectomy group. There were no statistical differences between the two groups in age, menstrual status, histological grade, pathological type, molecular subtypes, and NAC regimen (**Table 1**).

**Table 1:** Patient- and treatment-related characteristics.

Characteristics	All patients (n=585)	BCS(n=106)	Mastectomy (n=479)	*p* value
Age mean(y, range)	47.6(23-7)	46.2(33-62)	47.8(23-7)	0.075
Menopause				
No	375(64.1)	68(64.2)	307(64.1)	0.991
Yes	210(35.9)	38(35.8)	172(35.9)	
Multifocal/multicentric lesions				
No	404(69.1)	90(84.9)	314(65.5)	<0.001
Yes	181(30.9)	16(15.1)	165(34.5)	
Clinical T stage (pre-NAC)				
T1	37(6.3)	18(16.9)	19(4.0)	<0.001
T2	305(52.1)	69(65.1)	236(49.3)	
T3	116(19.8)	8(7.6)	108(22.5)	
T4	127(21.7)	11(10.4)	116(24.2)	
Clinical lymph node status(pre-NAC)				
Negative	118(20.2)	53(50.0)	65(13.6)	<0.001
Positive	467(79.8)	53(50.0)	414(86.4)	
Histological grade				
Grade 1-2	358(61.2)	71(67.0)	287(59.9)	0.177
Grade 3	227(38.8)	35(33.0)	192(40.1)	
Pathological type				
Invasive ductal carcinoma	559(95.6)	103(97.2)	456(95.2)	0.601
Other	26(4.4)	3(2.8)	23(4.8)	
ER status				
Negative	206(35.2)	46(43.4)	160(33.4)	0.051
Positive	379(64.8)	60(56.6)	319(66.6)	
PR status				
Negative	238(40.7)	56(52.8)	182(38.0)	0.005
Positive	347(59.3)	50(47.2)	297(62.0)	
HER-2 status				
Negative	319(54.5)	76(71.7)	243(50.7)	<0.001
Positive	266(45.5)	30(28.3)	236(49.3)	
Ki-67 index				
≤14%	35(6.0)	7(6.6)	28(5.8)	0.766
>14%	550(94.0)	99(93.4)	451(94.2)	
Molecular subtypes				0.335
Luminal A	31(5.3)	5(4.7)	26(5.4)	
Luminal B	369(63.1)	65(61.3)	304(63.5)	
HER-2	85(14.5)	12(11.3)	73(15.2)	
Triple negative	100(17.1)	24(22.7)	76(15.9)	
pCR				
No	471(80.5)	65(61.3)	406(84.8)	<0.001
Yes	114(19.5)	41(38.7)	73(15.2)	
M&P classification				
G1	33(5.6)	6(5.7)	27(5.6)	0.009
G2	137(23.4)	28(26.4)	109(22.8)	
G3	242(41.4)	29(27.4)	213(44.5)	
G4	61(10.4)	12(11.3)	49(10.2)	
G5	112(19.1)	31(29.2)	81(16.9)	
NAC regimens				
Anthracycline only	155(26.5)	28(26.4)	127(26.5)	0.733
Taxane only	103(17.6)	16(15.1)	87(18.2)	
Anthracycline+taxane	327(55.9)	62(58.5)	265(55.3)	
Endocrinotherapy				
No	205(35.0)	30(28.3)	175(36.5)	0.108
Yes	380(65.0)	76(71.7)	304(63.5)	

ER, estrogen receptor; PR, progesterone receptor; HER-2, human epidermal growth factor receptor 2; NAC, neoadjuvant chemotherapy; pCR, pathological complete response; M&P, Miller&Payne.

Among 106 patients in the BCS group, 61 patients (57.5%) underwent level I breast-conserving surgery, and 45 patients (42.5%) underwent level-II oncoplastic surgery. Intraoperative pathology revealed positive margins in 6.7% (7/106) of patients, and negative margins were obtained in all patients after resection. Among the 479 patients in the mastectomy group, 353 patients (73.7%) underwent modified radical mastectomy, 47 patients (9.8%) underwent mastectomy combined with sentinel lymph node biopsy, and 79 patients (16.5%) underwent modified radical mastectomy combined with breast reconstruction.

### Predictors of locoregional recurrence

During the follow-up period, the LRR rates were 11.3%(12/106) in BCS group and 16.3%(78/479) in mastectomy group, revealed no significant difference(*p* = 0.200). In patients who underwent BCS, univariate analysis showed that clinical T stage (pre-NAC), clinical lymph node status(pre-NAC), histological grade and pCR were factors to predict LRR. In multivariate analysis, positive clinical lymph node(HR 5.522, 95% CI 1.068-28.549, *p* = 0.042) and histological grade 3(HR 5.364, 95% CI 1.356-21.218, *p* = 0.017) remained independent factors for unfavorable LRR, and pCR predicted a better LRR (HR 0.114, 95% CI 0.013-0.981, *p* = 0.048) (**Table 2**).

**Table 2:** Factors predictive of locoregional recurrence in patients undergoing BCS after NAC.

Characteristic	Univariate analysis			Multivariate analysis	
	LRR (n=12)	No LRR (n=94)	*p* value	HR (95% CI)	*p* value
Age(years)			0.902		
≤35	3 (25.0%)	22(23.4%)			
>35	9 (75.0%)	73(76.6%)			
Multifocal/multicentric lesions			0.975		
No	10(83.3%)	80(85.1%)			
Yes	2(16.7%)	14(14.9%)			
Clinical T stage (pre-NAC)			0.013		
T1	1(8.3%)	17(18.1%)		Ref	
T2	5(41.7%)	64(68.1%)		0.772(0.061-9.699)	0.841
T3	1(8.3%)	7(7.4%)		0.625(0.024-16.471)	0.778
T4	5(41.7%)	6(6.4%)		3.648(0.239-55.644)	0.352
Clinical lymph node status(pre-NAC)			0.026		
Negative	2(16.7%)	51(54.3%)		Ref	
Positive	10(83.3%)	43(45.7%)		5.522(1.068-28.549)	0.042
Histological grade			0.014		
Grade 1-2	4(33.3%)	67(71.3%)		Ref	
Grade 3	8(66.7%)	27(28.7%)		5.364(1.356-21.218)	0.017
ER status			0.184		
Negative	3(25.0%)	43(45.7%)			
Positive	9(75.0%)	51(54.3%)			
PR status			0.161		
Negative	4(33.3%)	52(55.3%)			
Positive	8(66.7%)	42(44.7%)			
HER-2 status			0.788		
Negative	9(75.0%)	67(71.3%)			
Positive	3(25.0%)	27(28.7%)			
Ki-67 index			0.798		
≤14%	1(8.3%)	6(6.4%)			
>14%	11(91.7%)	88(93.6%)			
Molecular subtypes			0.708		
Luminal A	1(8.3%)	4(4.2%)			
Luminal B	9(75.0%)	56(59.6%)			
HER-2	0	12(12.8%)			
Triple negative	2(16.7%)	22(23.4%)			
pCR			0.049		
No	11(91.7%)	54(57.4%)		Ref	
Yes	1(8.3%)	40(42.6%)		0.114(0.013-0.981)	0.048
M&P classification			0.407		
G1	0	6(6.4%)			
G2	6(50.0%)	22(23.4%)			
G3	4(33.3%)	25(26.6%)			
G4	1(8.3%)	11(11.7%)			
G5	1(8.3%)	30(31.9%)			

ER, estrogen receptor; PR, progesterone receptor; HER-2, human epidermal growth factor receptor 2; LRR, locoregional recurrence; NAC, neoadjuvant chemotherapy; pCR, pathological complete response; M&P, Miller&Payne; HR, Hazard ratio.

In patients who underwent mastectomy, clinical T stage (pre-NAC), clinical lymph node status(pre-NAC), histological grade, molecular subtypes, pCR and MP classification were factors to predict LRR in univariate analysis. In multivariate analysis, positive clinical lymph node (HR 4.157, 95% CI 1.175-14.708, *p* = 0.027), histological grade 3(HR 3.919, 95% CI 2.114-7.267, *p* < 0.001), triple-negative disease(HR 12.719, 95% CI 5.427-29.809, *p* < 0.001), pCR (HR 0.177, 95% CI 0.014-0.948, *p* = 0.043), M&P classification G4(HR 0.090, 95% CI 0.014-0.583, *p* = 0.011) and G5 (HR 0.074, 95% CI 0.006-0.950, *p* = 0.046) were identified as independent predictors for LRR (**Table 3**).

**Table 3:** Factors predictive of locoregional recurrence in patients undergoing mastectomy after NAC.

Characteristic	Univariate analysis			Multivariate analysis	
	LRR (n=78)	No LRR (n=401)	*p* value	HR (95% CI)	*p* value
Age(years)			0.410		
≤35	8(10.3%)	55(13.7%)			
>35	70 (89.7%)	346 (86.3%)			
Multifocal/multicentric lesions			0.801		
No	50(64.1%)	264(65.8%)			
Yes	28(35.9%)	137(34.2%)			
Clinical T stage (pre-NAC)			<0.001		
T1	1(1.3%)	18(4.5%)		Ref	
T2	24(30.8%)	212(52.9%)		2.350(0.269-20.532)	0.440
T3	21(26.9%)	87(21.7%)		4.620(0.524-40.755)	0.168
T4	32(41.0%)	84(20.9%)		5.402(0.623-46.813)	0.126
Clinical lymph node status(pre-NAC)			0.012		
Negative	3(3.8%)	62(15.5%)		Ref	
Positive	75(96.2%)	339(84.5%)		4.157(1.175-14.708)	0.027
Histological grade(pre-NAC)			0.001		
Grade 1-2	32(41.0%)	255(63.6%)		Ref	
Grade 3	46(59.0%)	146(36.4%)		3.919(2.114-7.267)	<0.001
ER status			0.301		
Negative	30(38.4%)	130(32.4%)			
Positive	48(61.6%)	271(67.6%)			
PR status			0.106		
Negative	36(46.1%)	146(36.4%)			
Positive	42(53.9%)	255(63.6%)			
HER-2 status			0.915		
Negative	40(51.3%)	203(50.6%)			
Positive	38(48.7%)	198(49.4%)			
Ki-67 index			0.415		
≤14%	3(3.8%)	25(6.2%)			
>14%	75(96.2%)	376(93.8%)			
Molecular subtypes			<0.001		
Luminal A	2(2.6%)	24(6.0%)		Ref	
Luminal B	38(48.7%)	266(66.3%)		1.609(0.780-3.319)	0.198
HER-2	12(15.4%)	61(15.2%)		1.661(0.709-3.893)	0.242
Triple negative	26(33.3%)	50(12.5%)		12.719(5.427-29.809)	<0.001
pCR			0.005		
No	77(98.7%)	329(82.0%)		Ref	
Yes	1(1.3%)	72(18.0%)		0.177(0.014-0.948)	0.043
M&P classification			0.002		
G1	5(6.4%)	22(5.5%)		Ref	
G2	19(24.3%)	90(22.4%)		0.724(0.224-2.336)	0.589
G3	51(65.4%)	162(40.4%)		1.154(0.387-3.441)	0.797
G4	2(2.6%)	47(11.7%)		0.090(0.014-0.583)	0.011
G5	1(1.3%)	80(20.0%)		0.074(0.006-0.950)	0.046

ER, estrogen receptor; PR, progesterone receptor; HER-2, human epidermal growth factor receptor 2; LRR, locoregional recurrence; NAC, neoadjuvant chemotherapy; pCR, pathological complete response; M&P, Miller&Payne; HR, Hazard ratio.

### Disease-free and overall survival

**Figure 2:**
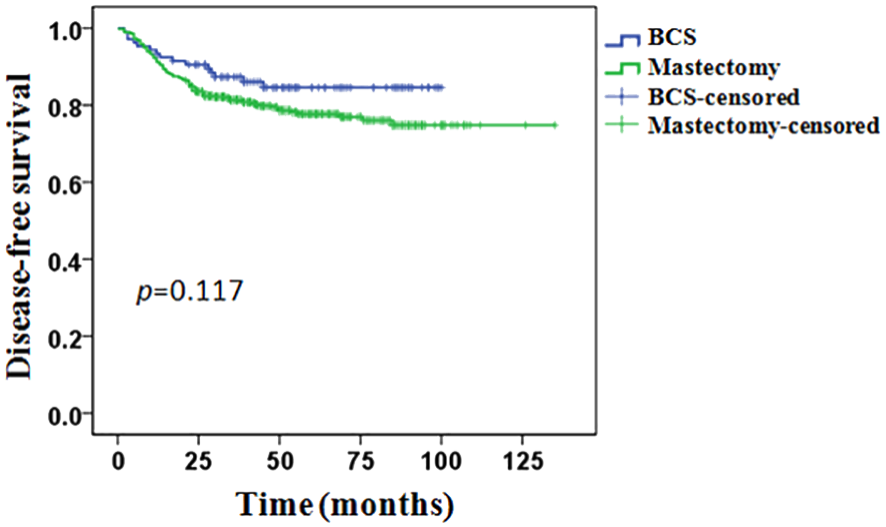
Disease−free survival in the BCS group compared with the mastectomy group.

The whole group of patients’ median follow-up time was 43 months (8–135months). In total, 116 (19.8%) patients experienced a recurrence or metastasis event: 15/106 (14.1%) in the BCS group and 101/479 (21.1%) in the mastectomy group. The 5-years DFS was 84.6% in the BCS group and 77.7% in the mastectomy group, however, the difference between the two groups was not statistically significant (*p* = 0.117, **Figure 2**).

**Figure 3:**
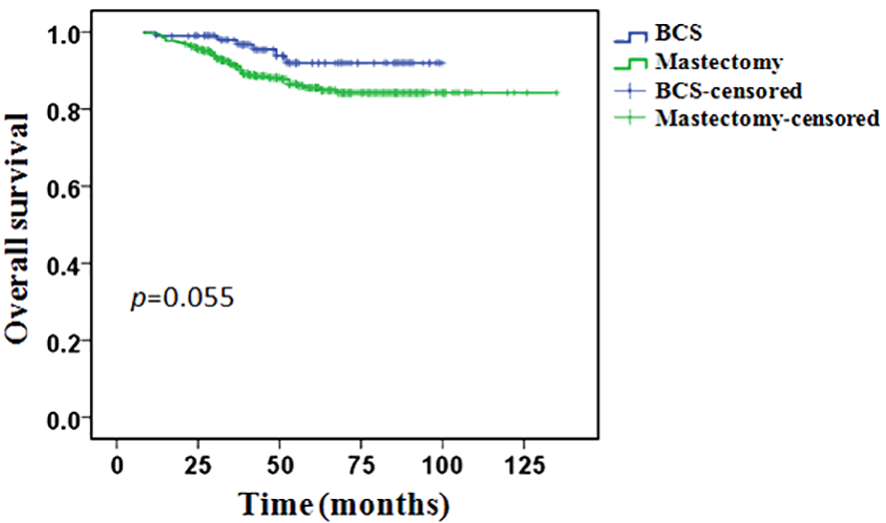
Overall survival in the BCS group compared with the mastectomy group.

During the follow-up period, 64 (10.9%) patients had died: 6/106 (5.7%) in the BCS group and 58/479 (12.1%) in the mastectomy group. In the BCS group, all patients’ death was related to breast cancer. In the mastectomy group, 55 (94.8%) patients’ death was related to breast cancer, 3 (5.2%) patients’ death was not associated with breast cancer. The 5-years OS was 92.0% in the BCS group and 85.5% in the mastectomy group, however, the difference between the two groups was not statistically significant (*p* = 0.055, **Figure 3**).

### Predictors of disease-free and overall survival

**Figure 4:**
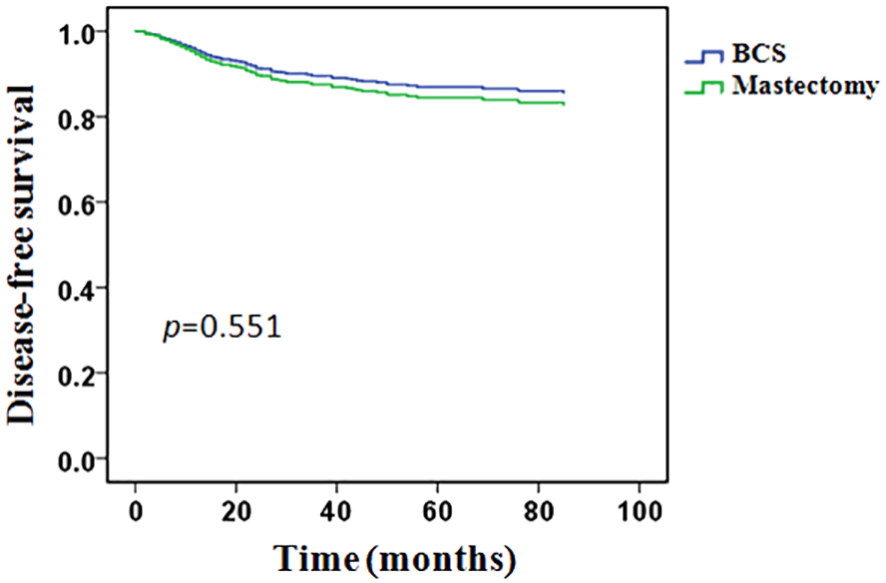
Adjusted survival functions for disease-free survival by type of breast surgery.

In univariate analysis, clinical T stage, clinical lymph node status, histological grade, PR status, molecular subtypes, pCR and M&P classification were significant variables related to DFS and included in the multivariable analysis, type of breast surgery was also included in the multivariable analysis as a candidate factor(*p* < 0.1). Multivariable Cox regression analysis showed that positive clinical lymph node (HR 1.808, 95% CI 1.057-3.416, *p* = 0.042), histological grade 3(HR 2.853, 95% CI 1.924-4.233, *p* < 0.001), triple-negative disease(HR 4.777, 95% CI 2.958-7.714, *p* < 0.001), pCR (HR 0.314, 95% CI 0.105-0.936, *p* = 0.038), M&P classification G4(HR 0.281, 95% CI 0.092-0.861, *p* = 0.026) and G5 (HR 0.312, 95% CI 0.089-0.917, *p* = 0.039) were independent predictors of DFS, but Clinical T stage and type of breast surgery were not independent predictors (**Table 4**). After adjusted for these significant variables, BCS had an HR of 0.828 (95%CI 0.468-1.460, *p* = 0.551), adjusted DFS functions are provided in **Figure 4**.

**Table 4:** Univariate and multivariate analysis of factors predicting DFS after NAC.

Characteristic	Univariate analysis		Multivariate analysis	
	HR(95%CI)	*p* value	HR(95%CI)	*p* value
Age(years)				
≤35	Ref			
>35	1.087(0.657-1.798)	0.745		
Multifocal/multicentric lesions				
No	Ref			
Yes	1.189(0.808-1.748)	0.380		
Clinical T stage(pre-NAC)				
T1	Ref		Ref	
T2	0.850(0.334-2.164)	0.734	0.755(0.289-1.974)	0.566
T3	1.746(0.671-4.549)	0.254	1.313(0.486-3.548)	0.592
T4	3.225(1.283-8.104)	0.013	1.965(0.756-5.106)	0.166
Clinical lymph node status(pre-NAC)				
Negative	Ref		Ref	
Positive	2.487(1.367-4.524)	0.003	1.808(1.057-3.416)	0.042
Histological grade				
Grade1-2	Ref		Ref	
Grade3	2.472(1.704-3.588)	<0.001	2.853(1.924-4.233)	<0.001
ERstatus				
Negative	Ref			
Positive	0.946(0.647-1.382)	0.773		
PRstatus				
Negative	Ref			
Positive	0.741(0.514-1.068)	0.108		
HER-2status				
Negative	Ref			
Positive	0.987(0.684-1.423)	0.943		
Ki-67index				
≤14%	Ref			
>14%	1.541(0.629-3.775)	0.345		
Molecular subtypes				
Luminal A	Ref		Ref	
Luminal B	1.015(0.622-1.659)	0.951	1.225(0.747-2.011)	0.422
HER-2	0.975(0.529-1.796)	0.935	1.083(0.583-2.010)	0.801
Triple negative	2.410(1.531-3.792)	<0.001	4.777(2.958-7.714)	<0.001
pCR				
No	Ref		Ref	
Yes	0.203(0.089-0.461)	<0.001	0.314(0.105-0.936)	0.038
M&P classification				
G1	Ref		Ref	
G2	1.005(0.442-2.283)	0.991	0.885(0.386-2.032)	0.774
G3	1.080(0.494-2.361)	0.847	0.811(0.366-1.798)	0.605
G4	0.408(0.137-1.215)	0.107	0.281(0.092-0.861)	0.026
G5	0.240(0.084-0.685)	0.008	0.312(0.089-0.917)	0.039
Type of breast surgery				
BCS	Ref		Ref	
Mastectomy	1.590(0.925-2.736)	0.094	0.974(0.540-1.754)	0.929

ER, estrogen receptor; PR, progesterone receptor; HER-2, human epidermal growth factor receptor 2; LRR, locoregional recurrence; NAC, neoadjuvant chemotherapy; pCR, pathological complete response; M&P, Miller&Payne; HR, Hazard ratio.

**Figure 5:**
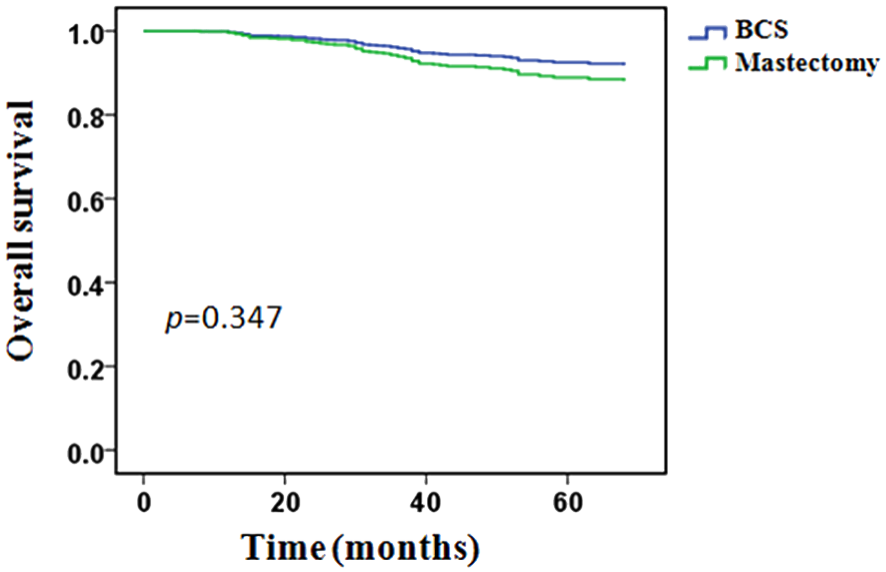
Adjusted survival functions for overall survival by type of breast surgery.

Univariate analysis showed that OS was related to clinical T stage, clinical lymph node status, PR status, molecular subtypes and pCR. Type of breast surgery was non-significant variables in univariate analysis, but also included in the multivariable analysis as a candidate factor(*p* < 0.1). Multivariable Cox regression analysis showed that positive clinical lymph node (HR 3.225, 95% CI 1.134-9.171, *p* = 0.028), triple-negative disease(HR 2.806, 95% CI 1.326-5.939, *p* = 0.007) and pCR (HR 0.224, 95% CI 0.068-0.736, *p* = 0.014) were independent predictors of OS, but clinical T stage, PR status and type of breast surgery were not independent predictors (**Table 5**). After adjusted for clinical lymph node status, histological grade, molecular subtypes and pCR, BCS had an HR of 0.662 (95%CI 0.280-1.564, *p* = 0.347), adjusted OS functions are provided in **Figure 5**.

**Table 5:** Univariate and multivariate analysis of factors predicting OS after NAC.

Characteristic	Univariate analysis		Multivariate analysis	
	HR(95%CI)	*p* value	HR(95%CI)	*p* value
Age(years)				
≤35	Ref			
>35	0.684(0.314-1.499)	0.343		
Multifocal/multicentric lesions				
No	Ref			
Yes	0.842(0.483-1.468)	0.545		
Clinical T stage(pre-NAC)				
T1	Ref		Ref	
T2	2.527 (0.340-18.788)	0.365	2.420(0.323-18.105)	0.390
T3	4.710 (0.619-35.821)	0.134	3.440(0.446-26.540)	0.236
T4	8.697 (1.183-63.926)	0.034	6.416(0.862-47.754)	0.070
Clinical lymph node status(pre-NAC)				
Negative	Ref		Ref	
Positive	4.223(1.534-11.623)	0.005	3.225(1.134-9.171)	0.028
Histological grade				
Grade1-2	Ref			
Grade3	1.351(0.872-2.208)	0.230		
ERstatus				
Negative	Ref			
Positive	0.702(0.428-1.150)	0.160		
PRstatus				
Negative	Ref		Ref	
Positive	0.598(0.366-0.978)	0.040	0.715(0.349-1.463)	0.358
HER-2status				
Negative	Ref			
Positive	0.861(0.524-1.413)	0.553		
Ki-67index				
≤14%	Ref			
>14%	2.175(0.532-8.894)	0.279		
Molecular subtypes				
Luminal A	Ref		Ref	
Luminal B	0.643(0.304-1.358)	0.247	0.662(0.312-1.403)	0.282
HER-2	0.939(0.418-2.110)	0.879	0.703(0.257-1.923)	0.493
Triple negative	2.727(1.529-4.865)	0.001	2.806(1.326-5.939)	0.007
pCR				
No	Ref		Ref	
Yes	0.204(0.064-0.650)	0.007	0.224(0.068-0.736)	0.014
M&P classification				
G1	Ref			
G2	1.152(0.334-3.981)	0.822		
G3	1.597(0.494-5.158)	0.434		
G4	0.163(0.017-1.571)	0.117		
G5	0.263(0.053-1.306)	0.102		
Type of breast surgery				
BCS	Ref		Ref	
Mastectomy	2.229(0.962-5.167)	0.062	1.277(0.528-3.089)	0.587

ER, estrogen receptor; PR, progesterone receptor; HER-2, human epidermal growth factor receptor 2; LRR, locoregional recurrence; NAC, neoadjuvant chemotherapy; pCR, pathological complete response; M&P, Miller&Payne; HR, Hazard ratio.

## Discussion

The present study included 585 patients with stage II-III breast cancer who received NAC followed by surgery. 479 patients underwent mastectomy after NAC, and 106 patients underwent BCS, with a breast-conserving rate of 18.1%. Among breast-conserving patients, the proportion of patients with a single lesion, early clinical tumor stage (pre-NAC) and lymph node negative was higher. Pathological complete remission from NAC was more favorable to accept BCS. Among patients in the BCS group, 57.5% patients underwent level I BCS, and 42.5% underwent level-II oncoplastic surgery. In patients whose tumors remain large after NAC, conventional BCS techniques may not result in satisfactory oncological and cosmetic outcomes, while oncoplastic surgery enables the resection of almost half of the existing breast tissue with good cosmetic results (22). In our study, there was no difference in age, pathological type, molecular subtype, histological grade, Ki-67 index and NAC regimens between the BCS and mastectomy groups, while Huynh V etal. reported that younger patients with significant mass tend to prefer mastectomy (23). In accordance with a few other cohort studies, our results reported that early-stage patients or patients who had a good clinical response from NAC were more suitable to BCS (24–26). This result suggested that although BCS rates reportedly increased overall after NAC, BCS was more likely to succeed in relatively early-stage breast cancer patients and who had a better response to NAC. However, after tumor downgrading through NAC, BCS presents a challenge for surgeons to accurately locate the lesion and completely remove the primary tumor. Accurate tumor localization is the key to obtain negative margin, there are recommended procedures as follows (11): careful local and systemic staging before NAC, use of the technique of breast tattooing or placement of clips before NAC to mark the primary tumor site. With the use of MRI staging evaluation, breast tattooing and placement of clips before NAC, accurate tumor location and pathologically negative margin were obtained in all breast-conserving patients in this cohort. It should be emphasized that for patients who achieved complete response on radiographic assessment, we performed resection in accordance with initial tumor size and margins marked before chemotherapy NAC. The improvement of plastic breast preservation technology allows us to remove larger breast tissue without compromising aesthetics, thereby ensuring a negative surgical margin.

The current study investigated locoregional recurrence rates, disease-free and overall survival after BCS compared with mastectomy in LABC patients having received NAC. In the BCS group, 15 patients (14.1%) had recurrence or metastasis, of which 12 (11.3%) had a local or regional recurrence, including ipsilateral breast and ipsilateral axilla. In the mastectomy group, 101 patients (21.1%) had recurrence or metastasis, of which 78 patients (16.3%) had a local or regional recurrence, including the ipsilateral chest wall, ipsilateral axilla, and ipsilateral supraclavicular lymph nodes. There was no statistically significant difference in LRR between the BCS group and the mastectomy group, indicating that BCS does not increase the risk of recurrence. For patients with LABC, the main challenge of undergoing BCS after NAC is that the local recurrence rate is acceptable compared to patients undergoing mastectomy. We observed that the risk of local recurrence in the breast-conserving group was significantly higher than the 2.1-4% risk of local recurrence reported in the literature for patients undergoing primary BCS before chemotherapy (27). But there was no increase in the local recurrence rate compared with the mastectomy group. It has been reported that patients with BCS after NAC have a higher risk of local recurrence than those who received primary BCS (28, 29). In NSABP B-18 and NSABP B-27 trials, LABC patients receiving NAC showed a higher local recurrence rate in the BCS group compared with the mastectomy group, and suggested that clinical tumor size, clinical node status, and treatment response were significant predictors of local recurrence after NAC (30). However, some recent retrospective studies failed to show a significant difference in local recurrence rate according to the type of surgery after NAC (10, 12). This is in concordance with our finding that BCS after NAC does not significantly increase the risk of locoregional recurrence compared to mastectomy. Previous studies (31, 32) demonstrated that DFS and OS rates were not statistically different between the BCS and the mastectomy groups. In our study, disease-free and overall survival appeared to be more favorable in patients with BCS than patients with mastectomy, but the difference was not statistically significant. These results suggest that BCS did not affect survival compared to mastectomy.

The prognosis of BCS after NAC is influenced by many factors, such as pathological features, primary tumor size, lymph node metastasis, chemotherapy response, and marginal condition. In the present study, we analyzed predictors of locoregional recurrence in different surgical subgroups. The results showed that in the BCS group, positive clinical lymph node, histological grade 3 and non-pCR were independent factors for unfavorable LRR. Moreover, positive clinical lymph node, histological grade 3, triple-negative disease and non-pCR were independent predictors of decreased disease-free survival and overall survival, M&P classification was also an independent predictor of disease-free survival. Apparently, the type of surgery did not affect the oncological outcome in patients treated with NAC. Some researchers have found that patients undergoing breast preservation after NAC have a higher local recurrence rate, possibly because the surgical edge is difficult to assess accurately (33). Our cohort did not analyze the correlation between surgical margin and prognosis because all breast-conserving patients obtained negative surgical margins. For breast cancer patients, the tumor stage and pathological pattern are the main indicators affecting the prognosis of patients, which has been reported in a large number of literature (31, 34, 35). By analyzing the SEER database, Sopik et al. found that the larger the initial tumor, the higher the probability of axillary lymph node metastasis, and the higher the risk of subsequent distant metastasis (36). When the tumor was less than 1cm, the risk of distant metastasis was only 0.5%, and when the tumor increased to 9-10 cm, the risk of distant metastasis was as high as 32.9%. They also found that the 15-year risk of breast cancer-related death increased with tumor size. However, tumor size was not a prognostic predictor in our study. This may be due to the small number of cases included, especially with fewer T1 tumors. In addition, our current follow-up time is relatively short for breast cancer patients and may interfere with the assessment of prognosis. It is well known that molecular typing of breast cancer is closely related to prognosis, and patients with triple-negative and HER-2 overexpression tend to have a poor prognosis. Lowery’s meta-analysis showed that HER-2 positive patients had a higher risk of local recurrence, which limited the application of breast-conserving surgery in HER-2 positive patients (37). A retrospective study has shown that triple-negative breast cancer was a negative predictor of disease-free survival in patients undergoing surgery after NAC (10). In our study, lymph node status and histological grade were independent predictors of locoregional recurrence in both BCS and mastectomy groups, and were also associated with disease-free survival and overall survival, which is consistent with previous reports. The only molecular subtype that seems to have effect on prognosis was triple-negative, presumably as a proportion of HER-2 overexpression patients treated with Herceptin. In addition, patients with early breast cancer usually have a long survival, and the median follow-up time of 43 months in this study is relatively short, the small number of events may affect the results of the analysis.

In addition to tumor stage and biological characteristics, chemotherapy response may be an important prognostic factor. Several retrospective studies have demonstrated that achieving pCR after NAC can result in better local control following surgery and benefit survival (38–40). The present study found that a good response to chemotherapy improves the breast-conserving rates of patients, and achieving pCR was a positive predictor for both LRR, DFS, and OS. A meta-analysis that included 12 international multicenter randomized controlled studies showed that achieving pCR after neoadjuvant chemotherapy improved patient outcomes regardless of molecular typing (39). This suggests that the response to chemotherapy after NAC in LABC patients, especially complete pathological response, may be a major factor in treatment decision and prognosis evaluation.

Finally, this study has some limitations that need to be addressed. First, it was a retrospective study from a single institution, which may result in selection bias. There are differences in some factors that may affect prognosis among patients in different surgical groups, such as tumor size, whether pCR after NAC, and patients primarily scheduled for mastectomy are more likely to have a poor prognosis to begin with. Second, we did not use any tool to estimate the sample size for this study. In addition, about one-third of the patients with positive HER-2 in our study did not use trastuzumab for economic reasons. The addition of trastuzumab would slightly decrease the overall recurrence rate. Finally, this study is also limited by the relatively short follow-up period.

## Conclusion

In summary, our study provide further insight into the long-term outcomes of BCS in patients treated with NAC, and this cohort represents real-world experience. To conclude, compared with mastectomy, BCS after NAC may not increase the risk of local recurrence or mortality, BCS can be performed in selected patients with small tumor size and good response to NAC.

## Data availability statement

The original contributions presented in the study are included in the article/supplementary material. Further inquiries can be directed to the corresponding authors.

## Ethics statement

The studies involving humans were approved by the Ethics Committee of Guangxi Medical University Cancer Hospital. The studies were conducted in accordance with the local legislation and institutional requirements. Written informed consent for participation was not required from the participants or the participants’ legal guardians/next of kin in accordance with the national legislation and institutional requirements.

## Author contributions

F-YL: Writing – original draft, Data curation, Formal analysis. ZM: Writing – review & editing, Methodology, Validation. BC: Data curation, Writing – review & editing. ZH: Writing – review & editing, Formal analysis. QM: Writing – review & editing, Project administration. QT: Project administration, Writing – review & editing, Funding acquisition, Writing – original draft.
